# Drink Tap: A Multisector Program to Promote Water Access and Intake in San Francisco Parks

**DOI:** 10.5888/pcd20.230007

**Published:** 2023-08-24

**Authors:** Margaret D. Rosenthal, Laura A. Schmidt, Roberto Vargas, Lauren S. Blacker, Charles E. McCulloch, Jeffery Ezennia, Anisha I. Patel

**Affiliations:** 1 School of Medicine, University of Alabama at Birmingham; 2 Philip R. Lee Institute for Health Policy Studies, University of California, San Francisco; 3 Center for Community Engagement, Clinical and Translational Science Institute, University of California, San Francisco; 4 Division of General Pediatrics, Stanford University School of Medicine, Palo Alto, California; 5 Department of Epidemiology and Biostatistics, University of California, San Francisco; 6 School of Medicine, University of California, Riverside

## Abstract

Taxes on sugar-sweetened beverages (SSBs), or drinks with added sugars, show promise in decreasing purchases and consumption of SSBs. Some have called for coupling such taxes with improvements in access to safe drinking water as a strategy for reducing inequities in SSB intake, yet no studies have examined such an approach. Drink Tap is a San Francisco-based program in which public tap water stations were installed in parks and public spaces (winter 2017) and promotional efforts (fall and winter 2018) encouraged water intake. At the same time, San Francisco and surrounding communities were also implementing SSB taxes. We conducted a quasi-experimental study to examine whether water access and promotion combined with SSB taxes affected beverage intake habits more than SSB taxes alone. We conducted 1-hour observations (N = 960) at 10 intervention parks (Drink Tap plus SSB taxes) and 20 comparison parks (SSB taxes only) in San Francisco Bay Area cities before (July–September 2016) and after (June–August 2019) implementation of Drink Tap. We found significant adjusted percentage increases in drinking water among visitors to intervention parks, compared with comparison parks: water from park water sources (+80%, *P* < .001) and water from reusable bottles (+40%, *P* = .02). We found no significant reductions in visitors observed drinking bottled water, juices, or SSBs. The Drink Tap intervention led to increases in water intake from park sources and reusable bottles across parks that surpassed increases achieved through SSB taxes alone. Jurisdictions should consider coupling tap water access and promotion with policies for reducing intake of SSBs.

**Summary**

**What is already known on this topic?**

Research on the effects of school-based water interventions found that increased access to safe, clean, and appealing tap water can increase water intake, decrease sugar-sweetened beverage intake, and prevent obesity. Evaluation of sugar-sweetened beverage taxes suggests that such tax policies decrease consumption of sugar-sweetened beverages.

**What is added by this report?**

The Drink Tap intervention, which combined access to and promotion of public tap water stations with existing sugar-sweetened beverage tax policies, led to increases in water intake; these increases surpassed increases achieved through sugar-sweetened beverage taxes alone.

**What are the implications for public health practice?**

Community-based interventions show promise to increase consumption of water, particularly in communities that implement complementary sugar-sweetened beverage tax policies.

## Introduction

Consumption of sugar-sweetened beverages (SSBs), or drinks with added sugars, is a risk factor for obesity, type 2 diabetes, and dental caries (1). Reducing SSB consumption and promoting access to tap water may have a beneficial impact on health (2). Increasing water consumption has health benefits, including improved physical performance, cognitive function, mood, and gastrointestinal and kidney function (3). Tap water, compared with bottled water, is cheaper and more likely to contain fluoride, which is important for dental health (3,4).

Consumption of water and SSBs involves health equity concerns. SSB intake is highest among low-income and racial and ethnic minority populations, specifically Mexican American and non-Hispanic Black people (5). Low-income and racial and ethnic minority populations are less likely than their White counterparts to consume tap water and more likely to purchase bottled water (5).

An increasing number of cities are adopting SSB taxes, which provide a financial disincentive to SSB consumption. Evaluation of the SSB tax in Berkeley, California, found a reduction in SSB consumption in low-income neighborhoods 4 months after implementation, and similar SSB tax evaluations suggest that such tax policies decrease purchases and consumption of taxed SSBs (6,7).

## Purpose and Objectives

The primary objective of this study was to describe how an intervention that combined access to and promotion of public tap water stations with existing SSB taxes, compared with SSB taxes alone, affected beverage intake habits in parks in low-income neighborhoods. Although school-based water promotion and access interventions were shown to increase water intake and decrease SSB intake among children (8,9), few studies have evaluated similar interventions in community spaces (10). The adoption of SSB taxes coupled with the Drink Tap water promotion and access program provided an opportunity to study the effect of increased water access in the context of newly implemented SSB taxes. We hypothesized that the addition of water access and promotion would increase water intake and reduce SSB intake to a greater extent than SSB taxes alone. Evaluation metrics included observations of beverage intake in parks and audits of water source conditions based on methods used in prior studies (11).

## Intervention Approach

The Drink Tap program was designed to increase access to, and promotion of, public tap water stations. In 2014, the San Francisco Health Improvement Partnership, a community health initiative supported by the Clinical and Translational Sciences Institute at the University of California, San Francisco, gathered community input showing that improved access to drinking water in public spaces was key to addressing racial and ethnic inequities in SSB intake (12). The San Francisco Health Improvement Partnership partnered with the San Francisco Public Utilities Commission to develop Drink Tap. The program consisted of installing reusable water bottle filling stations in winter 2017 in public schools, city parks, and recreation centers (Figure). It was implemented in San Francisco parks in low-income neighborhoods in which a large majority of residents are members of racial and ethnic minority groups and there is a high prevalence of chronic diseases such as obesity, type 2 diabetes, and dental caries, for which SSB intake is a risk factor. During the same period as the Drink Tap implementation (summer 2016 through summer 2019), SSB taxes went into effect in San Francisco (in January 2018) and neighboring city Oakland (in July 2017), offering a unique opportunity for conducting a quasi-experimental study to compare the effect of Drink Tap and SSB taxes in San Francisco communities with the effect of an SSB tax alone in Oakland. Following a recommendation from a community advisory board, San Francisco allocated 79% of its SSB tax revenue to health-related goals, including funding for Drink Tap (13). Community-based coalitions of nonprofit organizations serving African American, Asian American, and Latino communities led activities, including flyer distribution, health education sessions, and radio shows, that promoted water intake.

**Figure:**
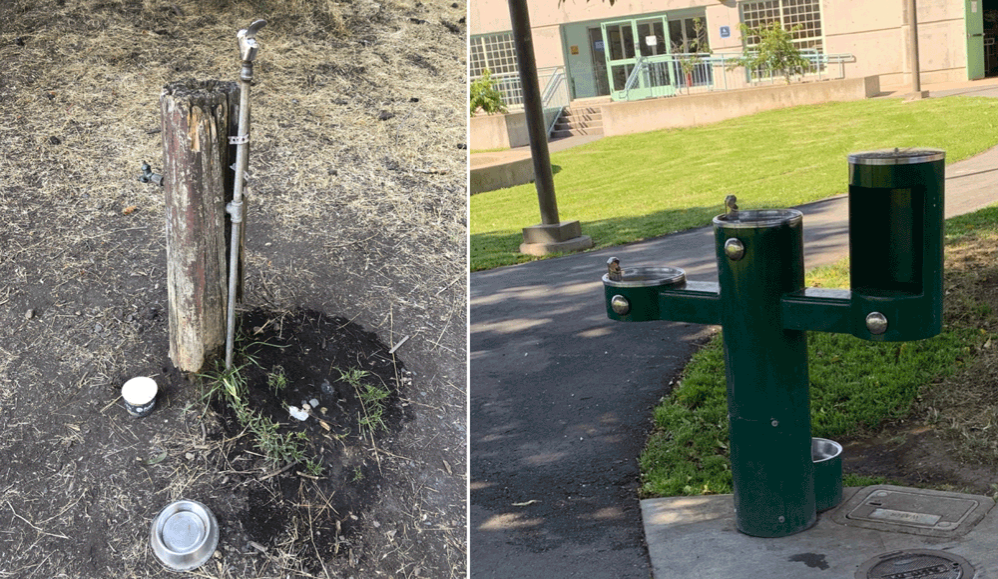
Drinking fountain in a comparison park (left) and Drink Tap water station in an intervention park (right). The Drink Tap water stations were installed in winter 2017.

## Evaluation Approach

The approach was a quasi-experimental study to examine how water stations in parks, water promotion, and SSB taxes, compared with SSB taxes alone, affected beverage consumption patterns among park visitors. For this study, we matched 10 parks in San Francisco that received Drink Tap stations with 10 comparison parks in San Francisco that did not receive Drink Tap stations and 10 comparison parks in Oakland that did not receive any drinking water improvements. Parks were matched by type of park amenities and income level and racial and ethnic composition of the neighborhood.

To examine changes in park visitors’ beverage consumption behaviors before and after implementation of Drink Tap interventions and SSB taxes, trained researchers conducted four 1-hour observations (9:30–10:30 am, 12:30–1:30 pm, 3:30–4:30 pm, and 6:30–7:30 pm) in each study park on 4 days (Monday, Wednesday, Friday, Saturday) in a single week. The intervention parks and matched control parks were observed on the same days in the same week. Researchers counted the total number of visitors in the designated park area, the total number of visitors who used the park water source, and any beverages park visitors had, categorized as plastic single-use water bottle, reusable bottle, coffee, milk, juice, nonalcoholic SSB, and alcoholic beverage. Baseline observations were conducted before installation of water stations, water education, and SSB tax implementation (July–September 2016), and follow-up observations were conducted after installations and promotion (June–August 2019). In intervention parks, the water source observed at baseline was the water source being replaced, and at follow-up the water source observed was the new station. In control parks with multiple water sources, the water source observed was in a location most similar to the location of the water source in the matched intervention park. In addition, researchers conducted audits to assess water source conditions. They categorized the type of drinking water source (eg, drinking fountain, sink), rated the source flow rate (too high, satisfactory, too low, erratic, or none), noted the appeal of the water source (presence of water stains, gum, rust, grime, hair, trash, debris, bird excrement, food, mold, clogged drain, insects, bodily fluids, and/or other), and documented the presence of any obstructions restricting access to the drinking source such as trash cans and cleaning equipment (yes or no). The study protocol was approved by the institutional review boards at Stanford University and University of California, San Francisco.

We considered covariates that could influence patterns of beverage consumption, including time of day (observation period), ambient temperature (°F, recorded on mobile phone applications), the racial and ethnic composition of the neighborhood in which the park was located (percentage of residents who are African American, Asian or Pacific Islander, Hispanic or Latino, or non-Hispanic White), and poverty status. We treated poverty status as a categorical (yes/no) variable according to whether the percentage of the neighborhood living below the federal poverty level was higher than the overall percentage of city living below the federal poverty level. We also tabulated the overall percentages of neighborhood residents living below the federal poverty level. We used the US Census 2012–2016 American Community Survey to determine income levels and racial and ethnic composition based on the zip code for each neighborhood (14).

Data were double-entered into Research Electronic Data Capture (REDCap) after observations were completed. We used descriptive analyses to summarize main outcomes and covariates. We used χ^2^ and Fisher exact tests for bivariate analyses of categorical outcomes and *t* tests for bivariate analyses of continuous outcomes. Mixed effects linear regression models were used to examine the impact of Drink Tap interventions on changes in visitors’ beverage intake patterns from preintervention (July–September 2016) to postintervention (June–August 2019). Because of skewed distributions, the outcome (proportion of visitors observed drinking each beverage) was treated as a numeric outcome and log-transformed. We used a difference-in-difference technique to estimate the intervention effect on intervention parks by comparing the change in outcomes over time between the intervention and control parks. Models included the outcome (proportion of park visitors drinking each beverage) and interaction of intervention status (intervention vs control) and time (baseline vs follow-up data collection). These models included random effects for park, matched park, and day of the week to account for the matching as well as clustering of observations within parks and days. Adjusted models also controlled for time of observation, average ambient temperature, neighborhood racial and ethnic composition, and neighborhood poverty level. Results were exponentiated to derive the percentage change in beverage intake patterns across intervention status over time. We used similar methods to examine the change in water source conditions from preintervention to postintervention. We performed all analyses in StataSE version 15.1 (StataCorp LLC).

## Results

We conducted 960 observations (480 at baseline and 480 at follow-up) at the 30 park sites. Park types included skate parks, recreation centers and playgrounds, playgrounds, and general parks (Table 1). In San Francisco study park neighborhoods, residents’ poverty status and race and ethnicity generally matched the overall demographic characteristics of San Francisco (Table 2). In Oakland study park neighborhoods, residents’ demographic characteristics differed slightly from the overall demographic characteristics of Oakland: we found similar values for poverty status but lower proportions of African American and Hispanic or Latino residents and higher proportions of Asian or Pacific Islander and non-Hispanic White residents.

**Table 1:** Sociodemographic Characteristics, by Park Type and Site, of Park Neighborhoods in Drink Tap, a Multisector Program to Promote Water Access and Intake in San Francisco Parks, 2016–2019^a^

Matched group	Park type	Site	Below federal poverty level	Hispanic or Latino	African American	Asian or Pacific Islander	Non-Hispanic White
1	Skate park	San Francisco intervention	11.4	7.1	1.7	32.6	53.8
		San Francisco control	9.2	6.6	1.3	48.3	37.9
		Oakland control	7.4	9.5	15.9	14.9	52.5
2	Recreation center and playground	San Francisco intervention	5.1	6.8	0.9	10.0	77.7
		San Francisco control	11.4	7.1	1.7	32.6	53.8
		Oakland control	7.5	7.7	6.7	14.9	64.2
3	General park	San Francisco intervention	20.4	22.3	28.3	36.6	7.9
		San Francisco control	11.8	24.2	7.4	56.5	9.4
		Oakland control	25.2	19.9	18.6	39.0	18.1
4	Playground	San Francisco intervention	12.1	35.7	3.3	13.7	42.6
		San Francisco control	12.1	35.7	3.3	13.7	42.6
		Oakland control	27.5	51.9	15.9	18.5	9.4
5	General park	San Francisco intervention	21.2	14.8	7.3	41.7	30.2
		San Francisco control	21.2	14.8	7.3	41.7	30.2
		Oakland control	11.4	15.7	16.5	20.5	40.1
6	General park	San Francisco intervention	11.8	24.2	7.4	56.5	9.4
		San Francisco control	11.8	24.2	7.4	56.5	9.4
		Oakland control	25.2	19.9	18.6	39.0	18.1
7	General park	San Francisco intervention	9.4	10.2	5.4	10.8	68.4
		San Francisco control	13.5	10.5	4.0	25.9	55.5
		Oakland control	4.7	7.8	5.3	13.0	68.0
8	General park	San Francisco intervention	13.1	8.1	10.8	24.7	52.4
		San Francisco control	10.9	11.2	5.2	29.2	49.5
		Oakland control time 1^b^	29.9	14.3	32.1	28.6	20.4
		Oakland control time 2^b^	11.4	15.7	16.5	20.5	40.1
9	Playground	San Francisco intervention	10.9	11.2	5.2	29.2	49.5
		San Francisco control	6.1	8.9	1.8	12.0	72.5
		Oakland control	16.0	14.3	25.6	8.6	44.7
10	Playground	San Francisco intervention	9.7	6.8	1.9	53.0	33.7
		San Francisco control	9.4	28.5	3.5	49.7	15.1
		Oakland control	29.9	14.3	32.1	28.6	20.4

a All values are percentages. Data from US Census 2012–2016 American Community Survey were used to determine income levels and racial and ethnic composition for each neighborhood (14). Federal poverty level is a national threshold defined by the US Census Bureau, varying by size of family and age of members.

b Oakland control park for group 8 was changed from time point 1 to time point 2 because of safety concerns for data collection at the original site.

**Table 2:** Comparison of Sociodemographic Characteristics of Park Neighborhoods and Cities in Drink Tap, a Multisector Program to Promote Water Access and Intake in San Francisco Parks, 2016–2019^a^

Characteristic	San Francisco, %		Oakland, %	
	Study park neighborhoods	City overall	Study park neighborhoods	City overall
Below federal poverty level	12.1	12.5	17.5	20.0
Race and ethnicity				
African American	5.8	5.1	18.0	24.1
Asian or Pacific Islander	33.7	33.8	22.2	16.4
Hispanic or Latino	15.9	15.3	17.5	26.7
Non-Hispanic White	40.1	41.2	36.7	27.3

a Data from US Census 2012–2016 American Community Survey were used to determine income levels and racial and ethnic composition based on the zip code for each neighborhood (14). Federal poverty level is a national threshold defined by the US Census Bureau, varying by size of family and age of members.

We found a significantly greater proportion of visitors drinking water from a park water source in intervention parks, compared with control parks, from preintervention to postintervention (+2.6 percentage points, *P* < .001) (Table 3). The adjusted ratio of the proportional changes in visitors drinking water from a park water source between intervention and control parks was 1.8, an 80% increase (27.4% increase in intervention parks and a 28.6% decrease in control parks [*P* < .001]).

**Table 3:** Changes in Proportion of Park Visitors Observed Drinking Beverages in 30 Study Parks Before and After Drink Tap Intervention and Sugar-Sweetened Beverage Taxes, 2016–2019^a^

Water source or type of drink	Baseline unadjusted mean, % (SD)	Follow-up unadjusted mean, % (SD)	PPD	*P *value b	Adjusted % change, race/ethnicity (95% CI)^c^	Ratio of adjusted trends^c^	*P* value^d^	Adjusted % change, race/ethnicity and poverty level (95% CI)^c^	Ratio of adjusted trends^c^	*P* value e
**Water from park water source**										
Intervention	5.6 (6.8)	7.1 (7.3)	+1.5	<.001	27.7 (–0.2 to 63.4)	1.8	<.001	27.4 (–0.1 to 62.5)	1.8	<.001
Control	4.5 (7.0)	3.4 (6.0)	–1.1		–29.4 (–40.8 to –15.9)			–28.6 (–40.0 to –15.1)		
DID	—	—	+2.6	—	—	—	—	—	—	—
**Reusable bottles**										
Intervention	4.6 (5.5)	9.2 (8.0)	+4.6	.02	84.5 (42.6 to 138.6)	1.5	.02	84.5 (43.2 to 137.8)	1.4	.02
Control	5.4 (7.9)	7.4 (8.5)	+2.0		26.5 (5.3 to 51.9)			28.1 (6.9 to 53.6)		
DID	—	—	+2.6	—	—	—	—	—	—	—
**Bottled water**										
Intervention	3.5 (4.8)	3.2 (5.2)	–0.3	.36	–11.8 (–28.9 to 9.3)	0.88	.33	–11.9 (–28.9 to 9.1)	0.88	.31
Control	3.4 (5.9)	3.6 (5.5)	+0.2		0.1 (–14.1 to 16.7)			0.6 (–13.7 to 17.2)		
DID	—	—	–0.5	—	—	—	—	—	—	—
**Water from any source**										
Intervention	13.8 (11.0)	19.5 (12.9)	+5.7	.02	38.0 (9.9 to 73.4)	1.4	.02	37.3 (12.5 to 67.5)	1.2	.07
Control	13.3 (13.8)	14.5 (12.5)	+1.2		0.1 (–14.9 to 17.8)			10.2 (–4.5 to 27.3)		
DID	—	—	+4.5	—	—	—	—	—	—	—
**Sugar-sweetened beverages**										
Intervention	1.9 (3.2)	1.7 (3.5)	–0.2	.87	–8.2 (–24.5 to 11.6)	1.0	.87	–8.6 (–24.8 to 11.1)	1.0	.95
Control	2.5 (4.4)	2.5 (5.5)	0		–9.9 (–21.7 to 3.5)			–9.3 (–21.0 to 4.2)		
DID	—	—	–0.2	—	—	—	—	—	—	—
**Juice**										
Intervention	0.3 (1.0)	0.3 (1.2)	0	.77	–3.0 (–12.3 to 7.3)	1.0	.78	–3.0 (–12.3 to 7.3)	1.0	.78
Control	0.5 (1.4)	0.3 (1.0)	–0.2		–4.7 (–11.3 to 2.4)			–4.6 (–11.3 to 2.5)		
DID	—	—	+0.2	—	—	—	—	—	—	—

Abbreviations: —, not applicable; DID, difference in difference; PPD, percentage-point difference.

a 52 water sources at baseline, 64 at follow-up.

b Mixed effects regression models used to examine intervention impacts on changes in beverage consumption in parks from baseline to follow-up, accounting for park type, date, and clustering of observations in parks.

c Ratio of trends is the ratio of the proportional changes for each group. For example, 1.8 is 1.277 (from a 27.7% increase) divided by 0.706 (from a 29.4% decrease).

d Mixed effects regression models used to examine intervention impacts on adjusted changes in beverage consumption in parks from baseline to follow-up, accounting for park type, date, and clustering of observations in parks and adjusting for time of day, ambient temperature during observation, and race/ethnicity of park neighborhood.

e Mixed effects regression models used to examine intervention impacts on adjusted changes in beverage consumption in parks from baseline to follow-up, accounting for park type, date, and clustering of observations in parks and adjusting for time of day, ambient temperature during observation, race/ethnicity of park neighborhood, and poverty level.

We found a significant increase in the proportion of park patrons drinking water from reusable bottles in intervention parks, compared with control parks, from preintervention to postintervention (+2.6 percentage points, *P* = .02). These findings remained in adjusted analyses: the adjusted ratio of the proportional changes for visitors drinking water from reusable bottles from preintervention to postintervention between intervention and control parks was 1.4, signifying a 40% increase (84.5% increase in intervention parks and a 28.1% increase in control parks [*P* = .02]). We observed no significant intervention effects for intake of bottled water, SSBs, or juice. 

From preintervention to postintervention, we found a greater proportion of visitors drinking water from any source in intervention parks, compared with control parks (+4.5 percentage points, *P* = .02). The adjusted ratio of the proportional changes for visitors drinking water from any source from preintervention to postintervention between intervention and control parks is 1.2, signifying a 20% increase (37.3% increase in intervention parks and a 10.2% increase in control parks [*P* = .07]). Although significance did not persist with full covariate adjustment for both neighborhood poverty level and racial and ethnic composition, the increase suggests public health significance.

Changes in water source conditions were not significant but were sizeable. Intervention water sources in poor condition decreased from 50.0% (8 of 16) to 43.3% (13 of 30), a 6.7 percentage-point decrease, and control water sources in poor condition increased from 65.7% (23 of 35) to 78.8% (26 of 33), a 13.1 percentage-point increase (*P* = .33).

## Implications for Public Health

We found that the Drink Tap intervention increased intake of water in parks from park water sources and reusable water bottles. These increases were likely driven by appreciable improvements in water source conditions in intervention parks. Increases in visitors drinking specifically from park water sources in intervention parks, compared with control parks, provide evidence for the success of the Drink Tap intervention. We observed no changes in SSB consumption among park visitors, however. To address SSB consumption, future work could include developing promotional campaigns that emphasize water as a substitute for SSBs and posting results of public drinking water quality tests to increase trust in tap water quality. We found appreciable, but not significant, decreases in poor water source conditions in intervention parks, even years after installation of Drink Tap stations. A previous evaluation of installation of water stations in Philadelphia recreation centers found improvements in water source conditions, and the relative lack of maintenance needed for the new stations over time offset the installation cost (15).

Our study had several limitations because of the quasi-experimental design. Our difference-in-difference design makes a parallel trend assumption that rates of change are expected to be the same in control and intervention sites. Although we would expect the rates of change to be similar, the similarity is not verifiable from data. Parks were matched to controls as closely as possible, and adjusted models in the analysis controlled for variables, including park neighborhood racial and ethnic composition, neighborhood poverty level, time of day of observation, and ambient temperature. However, the race and ethnicity of residents differed considerably between park neighborhoods in San Francisco and Oakland, reflecting the overall racial and ethnic composition of the cities. Because the intervention was not randomized, residual confounding may exist because of unmeasured confounders or inadequate adjustment.

Strengths of this study are that it was informed by community needs and evaluated a community effort. Although expansion of interventions to discourage intake of SSBs may be needed to decrease SSB consumption, community-based interventions such as Drink Tap show promise to increase water intake. In communities that implement SSB taxes, SSB tax revenue can be invested back into community interventions that promote water intake and increase water access.
